# Non-Invasive Strategies for Remineralization and Hypersensitivity Management in Molar–Incisor Hypomineralization—A Systematic Review and Meta-Analysis

**DOI:** 10.3390/jcm13237154

**Published:** 2024-11-26

**Authors:** Bianca Golzio Navarro Cavalcante, Éva Mlinkó, Bence Szabó, Brigitta Teutsch, Péter Hegyi, János Vág, Orsolya Németh, Gábor Gerber, Gábor Varga

**Affiliations:** 1 Centre for Translational Medicine, Semmelweis University, 1085 Budapest, Hungary; biancagolzio@hotmail.com (B.G.N.C.); evamlinko@gmail.com (É.M.); szabobence.tmk@gmail.com (B.S.); teutschbrigitta@gmail.com (B.T.); hegyi2009@gmail.com (P.H.); vag.janos@semmelweis.hu (J.V.); nemeth.orsolya@semmelweis.hu (O.N.); gerber.gabor@semmelweis.hu (G.G.); 2 Department of Oral Biology, Semmelweis University, 1089 Budapest, Hungary; 3 Department of Paediatric Dentistry and Orthodontics, Semmelweis University, 1088 Budapest, Hungary; 4 Department of Radiology, Medical Imaging Centre, Semmelweis University, 1083 Budapest, Hungary; 5 Institute for Translational Medicine, Medical School, University of Pécs, 7623 Pécs, Hungary; 6 Institute of Pancreatic Diseases, Semmelweis University, 1085 Budapest, Hungary; 7 Department of Restorative Dentistry and Endodontics, Semmelweis University, 1088 Budapest, Hungary; 8 Department of Community Dentistry, Semmelweis University, 1088 Budapest, Hungary; 9 Oral Morphology Group, Department of Anatomy, Histology and Embryology, Semmelweis University, 1085 Budapest, Hungary

**Keywords:** calcium phosphates, CPP-ACP, dental enamel hypomineralization, dentin sensitivity, fluorides, tooth remineralization agents

## Abstract

**Background:** Molar–incisor hypomineralization (MIH) is an enamel defect affecting molars and incisors, often leading to hypersensitivity, enamel breakdown, and increased caries risk. Non-invasive treatments, such as casein phosphopeptide–amorphous calcium phosphate (CPP-ACP) and fluoride varnish, show potential in remineralizing affected enamel and reducing sensitivity, but their efficacy is still debated. This study systematically reviews and analyzes the effectiveness of CPP-ACP and other non-invasive agents in improving remineralization and reducing hypersensitivity in MIH-affected teeth. **Methods:** A systematic search was conducted on PubMed, Embase, and Central in July 2024, including interventional and observational studies on remineralization and hypersensitivity in pediatric MIH patients (<18 years). A total of 1566 studies were found, with 15 included in the meta-analysis. A random-effects model was applied, including subgroup analysis by lesion severity. **Results:** CPP-ACP showed no statistically significant advantage over fluoride in remineralization (MD −3.80, 95% CI: −8.57; 0.98), but it significantly reduced hypersensitivity compared to fluoride varnish (MD −2.36, 95% CI: −3.83; −0.89). Although this reduction in hypersensitivity may be clinically relevant, the high heterogeneity (I² = 83%) and wide confidence intervals limit the reliability of these findings. **Conclusions:** CPP-ACP has a moderate effect in reducing hypersensitivity but does not outperform fluoride in remineralization. Other agents, such as calcium glycerophosphate and silver diamine fluoride, showed mild benefits. The current evidence base is limited and heterogeneous, highlighting the need for high-quality, long-term studies to confirm these findings and guide MIH management.

## 1. Introduction

Molar–incisor hypomineralization (MIH) is a qualitative developmental enamel defect affecting one or more permanent molars, often involving incisors [1,2,3,4]. Clinically, MIH appears as demarcated opacities, from creamy-white in mild cases to yellow-brown in severe cases. Severe lesions are commonly associated with post-eruptive enamel breakdown (PEB), susceptibility to caries, hypersensitivity, and aesthetic issues, which can potentially affect patients’ socio-psychological well-being and quality of life [5,6]. Globally, MIH affects approximately 13% of the population, and 27% of MIH-affected teeth have required or will require treatment, highlighting the urgent need for clear treatment protocols to address these issues [7,8,9,10]. MIH-affected enamel has decreased mineral density, hardness, and elastic modulus, as well as increased protein content and porosity, making it more susceptible to breakdown under masticatory forces, further complicating both treatment and prognosis [11,12,13]. Severely hypomineralized molars are at ten times greater risk of developing caries [14].

Dentin hypersensitivity (DH) affects 45% of patients with MIH worldwide [15], particularly in severe cases [16,17]. Hypersensitivity occurs when exposed dentinal tubules respond to external stimuli, such as thermal or tactile stimuli, triggering pain receptors [18,19]. Managing DH in MIH-patients is challenging due to their increased susceptibility to plaque accumulation and caries, along with their compromised oral hygiene caused by the altered properties of MIH-affected enamel [20]. Furthermore, additional challenges, such as pain, anesthetic difficulties, dental anxiety, and behavior management issues, negatively impact their quality of life and make clinical management complex [21,22,23,24].

Non-invasive treatments focusing on prevention are crucial for MIH, given the aesthetic, functional, and psychosocial concerns associated with hypersensitivity, PEB, and caries susceptibility [7]. The variable clinical presentation of MIH requires a wide range of treatment modalities, complicating clinical decision-making. A balanced approach integrating preventive care with symptom management and restorative measures is particularly important during the early developmental stages, when defective teeth are most susceptible to caries and breakdown [2,11,25,26]. Early preventive strategies, including remineralizing agents, aim to address clinical gaps by increasing the mineral content of hypomineralized enamel, reducing the risk of breakdown and caries progression, and managing hypersensitivity [27,28], especially in cases where more invasive treatments would not be appropriate.

Among the available non-invasive treatments, casein phosphopeptide–amorphous calcium phosphate (CPP-ACP) enhances remineralization by stabilizing calcium and phosphate ions, promoting their deposition on enamel lesions [29,30,31]. Fluoride varnish, another preventive agent, increases caries resistance and alleviates hypersensitivity by forming a temporary protective layer that slows demineralization and enhances fluoride uptake [32]. However, although these treatments show potential, more robust clinical evidence is needed to confirm their efficacy [33,34].

Emerging treatments with promising results, such as silver diamine fluoride (SDF), are also being explored to manage MIH-related sensitivity [35], although further studies are required to assess their long-term effectiveness [36]. Previous systematic reviews [6,11] suggest that remineralization using CPP-ACP may reduce mild or moderate hypersensitivity, and studies [10] have shown similar effects with other treatments such as fluoride varnish and 8% arginine with calcium carbonate. However, the previous reviews had small sample sizes and a limited number of studies and interventions, with no meta-analytic approach to investigate the impact of these interventions, emphasizing the need for an updated and more comprehensive analysis.

Despite considerable research over the past two decades, a definitive clinical protocol for the management of MIH remains unestablished [6]. The recent European Academy of Pediatric Dentistry (EAPD) guidelines offer only conditional strength of recommendations for remineralization and hypersensitivity treatments, reflecting limitations in the quality and quantity of existing studies, and underscoring the need for further research [26]. These guideline limitations highlight gaps in current evidence and a continued need to develop standardized, evidence-based protocols for the management of MIH [6].

Therefore, this study aims to systematically review and analyze the effectiveness of CPP-ACP and other non-invasive strategies in reducing hypersensitivity and enhancing remineralization in MIH-affected teeth.

## 2. Materials and Methods

### 2.1. Protocol and Registration

The PRISMA protocol was followed, and this systematic review was conducted according to the PRISMA 2020 statement [37]—the checklist is available in Supplementary Table S1. The protocol was registered at the International Prospective Register of Systematic Reviews (PROSPERO) [38] under CRD42022321486.

### 2.2. Information Sources and Searches

MEDLINE (via PubMed), Embase, and the Cochrane Central Register of Controlled Trials (CENTRAL) were searched in July 2023 and updated on 8 July 2024. No new terms were added or modified. No filters or restrictions were used, to ensure a comprehensive search. The reference lists of relevant articles and records included were manually searched for further eligible records.

### 2.3. Search Strategy

The search strategy combined terms related to “molar incisor Hypomineralization” and current non-invasive strategies for MIH lesions. More details on the different strategies used for each database can be found in Supplementary Table S2.

### 2.4. Study Selection

EndNote X20.2 (v.7) software (Clarivate Analytics, Philadelphia, PA, USA) was used to manage records. After excluding duplicates, two independent authors (B.C. and É.M.) screened the remaining studies by title, abstract, and finally full text. Cohen’s kappa [39] coefficient was calculated to assess the agreement between B.C. and É.M. during the selection process. Any disagreements were resolved through discussion between the two reviewers, revisiting the eligibility criteria, consulting additional sources or, if the conflict persisted, consulting a third reviewer (G.V.).

### 2.5. Eligibility Criteria

Type of participants: Studies involving subjects under 18 years of age diagnosed with MIH according to any validated diagnostic criteria (EAPD, Ghanim, Weerheijm). Type of intervention/control: Any non-invasive interventions used for remineralization and hypersensitivity management, with at least one data point before and after the intervention. Primary outcome: Remineralization potential measured instrumentally through fluorescence-based methods: (1) Laser fluorescence (LF) to assess the degree of early carious lesions. (2) Quantitative light-induced fluorescence (QLF) to assess the degree of early caries regions compared to the surrounding healthy tooth structure. Secondary outcome: Hypersensitivity management measured instrumentally and visually through any validated pain outcome measure, such as the visual analog scale (VAS), SCASS, WBFS, and others.

### 2.6. Inclusion and Exclusion Criteria

Our inclusion criteria for the studies were as follows: (1) in vivo, clinical studies, and observational studies; (2) patients under 18 years diagnosed with MIH lesions; (3) receiving any non-invasive agents used for remineralization and hypersensitivity management. Our exclusion criteria were as follows: (1) in vitro studies, in situ studies, case reports, case series, and reviews; (2) associated developmental defects of enamel other than MIH (demarcated opacities, diffuse opacity, hypoplasia, amelogenesis imperfecta, dentinogenesis imperfecta, white spot lesions, erosion, fluorosis).

### 2.7. Data Extraction

The necessary correspondence was conducted with the studies’ authors to obtain the full texts when they were not available electronically. Two investigators (B.C. and É.M.) extracted data using the standardized data collection form, which included the following fields: title, first author, year of publication, countries, study design, number of patients and teeth, main findings, patient demographics, type of intervention, outcome measures for remineralization efficacy, and hypersensitivity. Subgroups were created according to lesion severity by the EAPD criteria (mild and severe) [26] when described (Supplementary Table S3). A third independent reviewer (G.V.) resolved disagreements when needed. Teeth were used as a unit measure for the quantitative analysis of both outcomes. In studies with multiple follow-up points, the data closer to 3 months were used for analysis.

### 2.8. Risk-of-Bias Assessment

The ROBINS-I tool [40] was used to assess non-randomized studies through seven domains: bias due to confounding, bias due to participant selection, bias in classification of interventions, bias due to deviations from intended interventions, bias due to missing data, bias in the measurement of outcomes, and bias in the selection of the reported results. For randomized controlled trials, the ROB 2 tool [41] was used, including five domains: bias from the randomization process, deviations from intended interventions, bias due to missing outcome data, bias in the measurement of the outcome, and bias in the selection of results reported.

Studies were generally judged to be at a “low” risk of bias if all domains were at low risk, at “moderate” risk of bias if all domains were at low or moderate, and at “serious” risk of bias if at least one domain was judged to be at severe risk. Two independent reviewers (B.C. and É.M.) conducted the assessment, and disagreements were first addressed through discussions and review of the study details. If a resolution could not be reached, a third reviewer (G.V.) was consulted to resolve the conflict.

### 2.9. Statistical Analysis

A random-effects model was used to pool effect sizes due to the expected substantial heterogeneity across studies in terms of interventions, outcome measures, and patient characteristics. The mean values and the differences between them (MD) were used as an effect size measure, with 95% confidence intervals (CIs). To calculate study MDs and pooled MDs, the sample size, the mean, and the corresponding standard deviation (SD) were extracted from each study. The mean values of the control group were subtracted from the mean values of the experimental group.

In the case of hypersensitivity, instead of a pooled SMD effect size, all different scales were converted to a common, more easily interpretable scale, ranging from 0 to 10, and the MD of this common scale was selected as the effect size. As for the VAS and the WBFS, this 0–10 was the original scale; these were not modified. The SCASS ranges from 0 to 3, and the VAS Pimenta ranges from 0 to 4, so the effect measures extracted in these units were multiplied by 3.33 and 2.5, respectively. In this way, all hypersensitivity measures could be pooled in a universally interpretable 0-to-10 scale. The limitation of this approach is that we are assuming that the relationship between scales is linear and that the conversion factor is applicable across all studies, meaning proportional changes to the 0–10 scale, which may not accurately capture subjective pain differences and could introduce potential bias.

The inverse variance weighting method was used to calculate the pooled mean and MD values. To estimate the heterogeneity variance measure (τ2), we used the restricted maximum-likelihood estimator with the Q profile method for confidence intervals [42,43]. The t-distribution-based method was used for the CI of MD calculations of individual studies. We used a Hartung–Knapp adjustment [44,45] for CIs.

For subgroup analyses, we used a fixed-effects “plural” model (also known as a mixed-effects model), assuming that all subgroups had a common τ2 value, as we did not expect substantial between-study heterogeneity differences across subgroups, and the number of studies was relatively small in some subgroups—as recommended by Borenstein et al., (2009) [46]. To assess the differences between the subgroups, we used a “Cochrane Q” test (an omnibus test) between subgroups [42]. The null hypothesis was rejected at a 5% significance level. Sensitivity analyses were performed to confirm the reliability of subgroup differences. This involved assessing whether excluding any study or variation in subgroup criteria would significantly alter the effect sizes.

Tests for funnel plot asymmetry are generally only used when there are at least ten studies in the meta-analysis, as the statistical power of such tests is low with fewer studies. Therefore, such tests could not be performed, due to the relatively low number of studies included in this study. Without sufficient studies, potential publication bias was assessed qualitatively by reviewing study characteristics, selective reporting of outcomes, and trial registries where available.

## 3. Results

### 3.1. Study Selection

**Figure 1:**
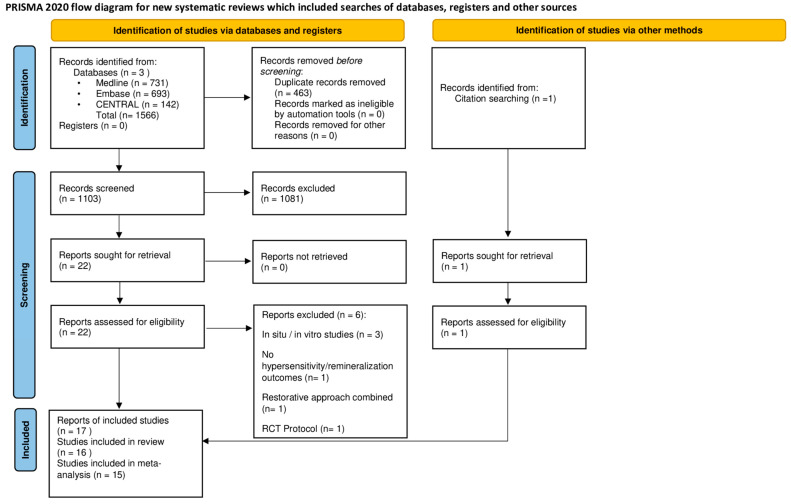
Preferred Reporting Items for Systematic Reviews and Meta-Analyses (PRISMA) flowchart showing the selection process.

Our systematic search yielded 1566 records, and after duplicate removal, 1103 remained. Their titles and abstracts were screened, and 22 items were found to be eligible and went through full-text selection. A total of 16 reports from 15 studies were included, and one more study was identified through the screening of references, making a total of 17 reports from 16 studies. Of these, 15 studies were included in the quantitative analysis. The PRISMA flow diagram summarizing all stages of the selection process is shown in Figure 1.

### 3.2. Study Characteristics

#### 3.2.1. Description of the Included Studies

The main characteristics of the included studies are presented in Table 1. A total of 15 studies were included, with 740 patients and 1997 teeth. All of the studies were published between 2016 and 2024 and were randomized trials, non-randomized trials, or prospective cohorts. The age of the population ranged from 3 to 17 years old. The follow-up timepoints of the studies ranged from 0.5 to 24 months. For the quantitative synthesis, a single data timepoint closer to 3 months was selected—within a range from 0.5 to 3 months—to allow for a more homogeneous analysis of treatment effects in a short-term follow-up.

#### 3.2.2. Description of the Excluded Studies

Six studies were excluded during full-text selection, for reasons specified in Supplementary Table S4.

Two studies were excluded from the meta-analysis: Restrepo and coinvestigators [47] used an outcome measure (QLF) different from the other studies (LF), preventing a direct comparison. Their results showed no favorable effect of fluoride varnish on the remineralization of anterior teeth. Erbas Unverdi and coworkers [35] reported a follow-up on the work of Ballikaya et al. [48], and no new quantitative data on hypersensitivity outcomes were available for further analysis. This study reported that no teeth showed hypersensitivity after the application of silver diamine fluoride (SDF) during 18 months of follow-up. Those studies were not included in the quantitative synthesis, to ensure the consistency and relevance of the outcome measures.

### 3.3. Risk of Bias Within Studies

Sixteen studies were assessed for the risk of bias. Of the four non-randomized studies assessed with the ROBINS-I tool (Supplementary Figure S1a), one was considered serious, three moderate, and none low-risk. Serious bias was present in the selection of reported results only for one study, whereas one was moderate, and two were at low risk. All four studies were at moderate risk of bias due to the selection of participants and at low risk due to deviations from intended interventions.

For the randomized controlled trials, 12 studies were assessed through the ROB 2 tool (Supplementary Figure S1b). Six studies were at high risk, five at moderate risk, and one at low risk of bias. The risk of bias due to deviations from the intended intervention domain was serious for five studies, moderate in five, and low in two. On the other hand, the risk of bias due to missing outcome data was serious in one study and low in eleven. Two domains were not assessed for serious risk of bias, including bias in the selection of the reported results (seven studies were assessed as moderate risk, and five as low risk) and bias from the randomization process (four at moderate risk, eight at low risk).

### 3.4. Results of Individual Studies and Synthesis of Results

Our meta-analysis included 15 studies, which provided sufficient data for the quantitative analysis.

### 3.5. Primary Outcome (Remineralization Potential)

#### 3.5.1. Laser Fluorescence (LF) Values

Six studies assessed the efficacy of remineralization by laser fluorescence. LF uses a relative numerical scale from 0 to 99 (LF DIAGNOdent cutoffs proposed by the manufacturer: 0–10, sound teeth; 10–30, enamel caries, >30, dentin caries). Mean values, expressed in forest plots, represent the difference in LF values between the baseline and a given timepoint, representing the change in mineral content. The more negative the value, the greater the remineralization. In the included studies, LF values were reported separately according to the lesion severity (mild or severe).

**Figure 2:**
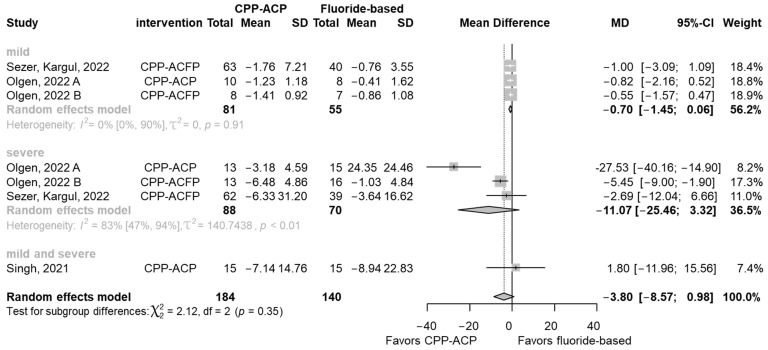
Forest plot comparing the CPP-ACP vs. fluoride-based groups, using the change from baseline LF values of mild and severe MIH lesions for remineralization [49,50,51].

No statistically significant differences were found in favor of the CPP-ACP group compared to the fluoride-based agents (MD −3.80, 95% CI: −8.57; 0.98), nor any clinically significant reduction in LF values (Figure 2). The χ² test results (χ² = 2.12, df = 2, *p* = 0.35) suggested that the studies could be combined without concern for high heterogeneity. However, the low number of studies may limit the ability to detect heterogeneity. Results were sub-grouped according to lesion severity. For mild lesions, despite the low heterogeneity, no statistically significant differences were found between the groups (MD −0.70, 95% CI: −1.45; 0.06, I^2^ = 0% [CI: 0%, 90%]). Severe lesions exhibited a mean difference (MD) of −11.07 (95% CI: −25.46; 3.32, I² = 83% [CI: 47%, 97%]) following treatment with CPP-ACP compared to the fluoride-based group. Although this reduction suggests a potentially clinically relevant effect on MIH lesions, the high heterogeneity (I² = 83% [CI: 47%, 97%]) and wide confidence intervals indicate substantial variability across studies. Therefore, these findings should be interpreted with caution.

##### Mild Lesions

**Figure 3:**
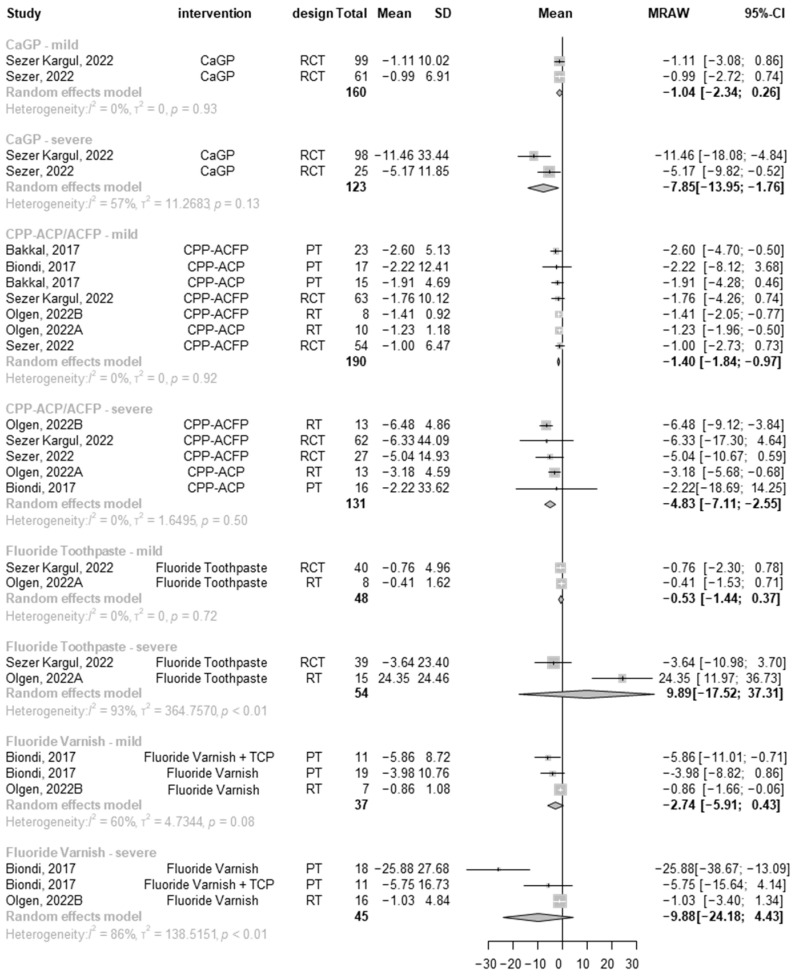
Forest plot presenting the mean change from baseline LF values with different agents used for the remineralization of mild and severe MIH lesions [49,50,52,53,54].

Five studies that reported mild MIH lesions were included, with 435 teeth in total (Figure 3). Four different interventions were analyzed in different subgroups: CaGP showed a reduction of −1.04 in the LF scale in 160 teeth (mean −1.04, 95% CI: −2.34; 0.26; I^2^ = 0), while CPP-ACP agents showed a reduction of −1.40 (mean −1.40, 95% CI: −1.84; −0.97; I^2^ = 0 [CI: 0%, 71%]) in 190 teeth. When fluoride toothpaste alone was used, a pooled mean reduction of −0.53 was found for 48 teeth (mean −0.53, 95% CI: −1.44–0.37; I^2^ = 0), while fluoride varnish reduced lesion severity by 2.74 (mean −2.74, 95% CI: −5.91–0.43; I^2^ = 60% [CI: 0%, 89%]) in 37 teeth.

##### Severe Lesions

Four studies reported severe MIH lesions and included 434 patients in total (Figure 3). Four different interventions were analyzed in different subgroups: CaGP showed a reduction of −7.85 in the LF scale for 123 teeth (mean −7.85, 95% CI: −13.95–−1.76; I^2^:57% [CI: 0%, 90%]), whereas CPP-ACP products showed a reduction of −4.83 (mean −4.83, 95% CI: −7.11; −2.55; I^2^:0% [CI: 0%, 79%]) in 131 teeth. When fluoride toothpaste alone was used, an actual increase of 9.89 in the severity of MIH lesions was found for 54 teeth (mean 9.89, 95% CI: −17.52–37.31; I^2^:93% [CI: 77%, 98%]), whereas fluoride varnish showed the most significant mean reduction in lesion severity (mean −9.88, 95% CI: −24.18; 4.43; I^2^:93% [CI: 60%, 95%]) for 45 teeth.

### 3.6. Secondary Outcome (Hypersensitivity)

**Figure 4:**
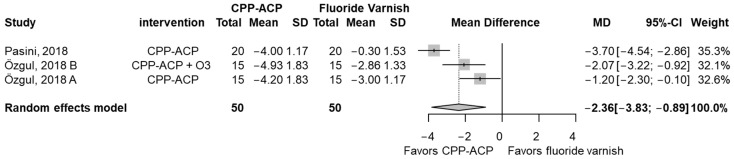
Forest plot comparing the CPP-ACP vs. fluoride varnish groups by changes in hypersensitivity compared to baseline, measured by the VAS pain scale (0 to 10), for MIH lesions [56,57].

Hypersensitivity was measured on different scales and then converted to a pain scale ranging from 0 to 10 [55]. CPP-ACP showed a statistically significant reduction in hypersensitivity compared to fluoride varnish (MD −2.36, 95% CI: −3.83; −0.89) (Figure 4). The MD (−2.36) indicates a clinically significant improvement.

**Figure 5:**
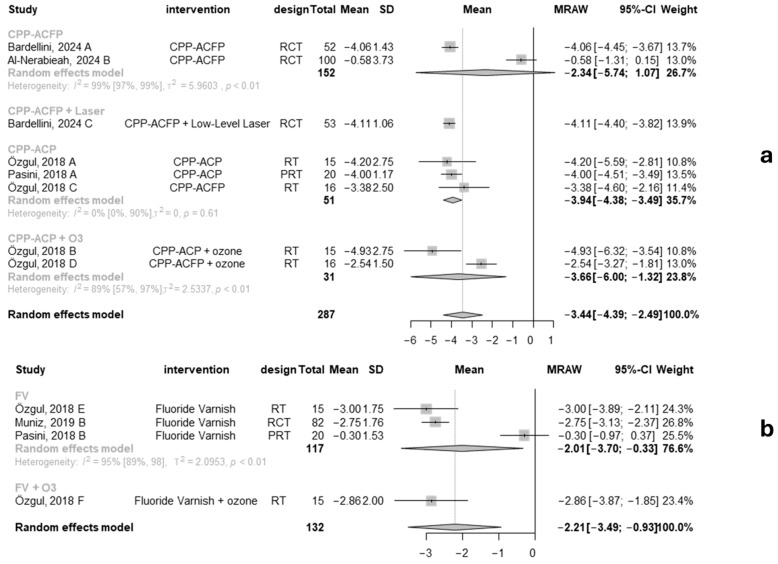
(**a**) Forest plot presenting CPP-ACP (subgroups by CPP-ACP type): mean change from baseline values for hypersensitivity measured on the VAS pain scale (0 to 10). (**b**) Forest plot presenting fluoride varnish (subgroups by FV): mean change from baseline values for hypersensitivity measured on the VAS pain scale (0 to 10) [56,57,58,59,60].

A subgroup analysis compared different CPP-ACP combinations (CPP-ACFP, CPP-ACFP and laser, CPP-ACP and ozone) (Figure 5a). CPP-ACP alone showed a reduction of 3.94 in hypersensitivity (mean −3.90, 95% CI: −4.46–−3.35; I^2^:0%; *p* = 0.62) among 51 patients. The combination of CPP-ACP with ozone had a similar effect (mean −3.66, 95% CI: −6.00–−1.32; I^2^:89%; *p* < 0.01). Overall, the pooled mean effect for CPP-ACP treatments was −3.44 (95%CI: −4.39; −2.49), indicating a clinically significant reduction in hypersensitivity for patients with MIH.

In contrast, three studies found a mean hypersensitivity reduction of 2.21 for fluoride varnish treatments (mean −2.21, 95% CI: −3.49–−0.93; I^2^:95%; *p* < 0.01) in 132 patients, with fluoride varnish combined with ozone showing a greater reduction (mean −2.86, 95% CI: −3.87; −1.85) (Figure 5b). CPP-ACP showed a more significant decrease in hypersensitivity and more clinically relevant results.

Six studies reported different interventions for hypersensitivity in MIH lesions (Supplementary Figure S2). Some interventions, such as amine fluoride toothpaste (mean −1.50, 95% CI: −3.28–0.28), arginine/calcium carbonate paste (mean −4.67, 95%CI:−6.22;−3.12), hydroxyapatite paste (mean −1.90, 95% CI: −4.56; 0.76), low-level laser treatment (mean −3.33, 95% CI: −4.47; −2.20), and silver diamine fluoride (mean −2.57, 95% CI: −6.36; −1.21), are displayed for visualization, as they were only included in one study. Among these, low-level laser treatment showed the largest reduction in hypersensitivity across 136 patients.

## 4. Discussion

Our study assessed the effectiveness of non-invasive treatments for molar–incisor hypomineralization (MIH), focusing on fluorescence-based remineralization and reduction in hypersensitivity. While CPP-ACP showed a modest improvement over fluoride-based treatments in terms of remineralization measured by LF and QLF, the difference was not statistically significant, indicating a potential clinically relevant advantage in mineral content. In addition to fluoride-based systems, CPP-ACP and calcium glycerophosphate are agents that are used to remineralize MIH lesion structures [10,27]. Topical applications of 10% CPP-ACP, CPP-ACFP, calcium glycerophosphate, 5% NaF varnish, and 5% NaF varnish with tricalcium phosphate were more effective for severe lesions [49,50,51,52,53,54,61]. In contrast, minimal improvement was observed in mild lesions. Fluoride toothpaste (1450 ppm) alone worsened the lesions over time [49,50]. This may relate to the higher mineral needs of severely affected enamel, which CPP-ACP, with its calcium and phosphate contents, may better address than fluoride alone. While both CPP-ACP and fluoride show benefits, CPP-ACP appears to be more suited for severe lesions.

There are accurate quantitative in vitro methods to measure the mineral content of enamel in MIH, such as X-ray microtomography (XMT) [62,63,64]. However, despite their quantitative precision, these methods are laboratory-based and are not applicable in daily clinical practice. Therefore, in the present meta-analysis of in vivo studies, non-invasive and validated tools such as LF and QLF were included [65,66,67]. LF devices, such as DIAGNOdent, measure the fluorescence emitted from carious or hypomineralized enamel and relate it to lesion severity. These values should be interpreted with caution, as they provide indirect measurements of remineralization, such as the presence of bacterial porphyrins in carious lesions [68] or increased protein levels for non-carious hypomineralized enamel [69]. In addition, factors such as moisture, plaque, and surface texture can affect readings [70,71]. One study has demonstrated a moderately strong correlation between LF scores and the mechanical properties of MIH-affected enamel [69]. Still, there is a lack of robust evidence for the relationship between LF and MIH severity. Accurate measurement of remineralization is challenging, as the reduction in LF or QLF readings observed during studies does not always correspond to a noticeable clinical improvement [26]. Therefore, LF results should still be combined with visual inspection and consideration of the symptoms of patients [70].

There is conflicting evidence on the efficacy of CPP-ACP and fluoride on remineralization. The present findings corroborate a recent meta-analysis by our group. We found that the effect of CPP-ACP was not superior to that of fluoride alone in remineralizing early carious lesions, and no difference was found in LF values between the groups, despite their relevant action [72]. Limited evidence is available on the effect of CPP-ACP on MIH. In contrast, in situ studies have indicated increased mineral content and physical strength after the topical application of CPP-ACP in MIH-affected enamel [34] and demonstrated that CPP-ACP can accelerate and enhance the maturation of enamel structure in MIH [73]. Kumar (2022) showed a reduction in the carbon content of hypomineralized enamel slabs and a consequent increase in Ca, P, and F after the application of CPP-ACP and fluoride varnish [33].

The clinical evidence supporting the use of fluoride for remineralization in MIH is limited, with only a few studies examining different fluoride formulations [27,33,47,50,53,74]. Still, topical FV is recommended to prevent caries in patients with MIH, along with regular dental checkups every 3-to-6 months and a healthy diet with controlled sugar intake [2,28]. In contrast, a longitudinal study demonstrated an increased risk of dental caries and susceptibility to enamel breakdown in molars affected by MIH, regardless of the application of fluoride varnish [75]. In our study, the progression of lesions in teeth severely affected by MIH that used only fluoride toothpaste coincided with the increased susceptibility to caries in severe MIH shown by previous studies—as severity increases, hypersensitivity occurs, and oral hygiene deteriorates [20,76]. Conflicting evidence on the efficacy of CPP-ACP and fluoride may arise from variations in study designs, sample characteristics, and even the severity of MIH lesions. Differences in formulations and application methods could also contribute, highlighting the need for standardized protocols.

Furthermore, a recent in vitro study [77] observed an enhanced remineralization effect in artificial carious lesions when CaGP was combined with conventional fluoride toothpaste. Similarly, an in situ study by Emerenciano and coinvestigators (2023) reported that the incorporation of nano-sized β-CaGP into toothpaste significantly increased the bioavailability of calcium and phosphate, resulting in a higher remineralization effect compared to fluoride toothpaste (1100 ppm) alone [78].

The evaluation of hypersensitivity in children is clinically challenging due to the subjective nature of their pain perception [15], which may be influenced by emotional, psychological, and environmental factors [20]. Tactile, cold, and evaporative air stimuli are commonly used and recommended for the induction of dentinal pain, as they are physiological and reproducible [79]. The Schiff Cold Air Sensitivity Scale (SCASS) is widely used to measure DH [80]. Face scales, such as the Wong–Baker FACES Pain Rating Scale (WBFPRS), are visual tools for measuring pain intensity, particularly in children. These scales display facial expressions, ranging from no pain (neutral expression) to severe pain (distressed expression), and patients select the face that best reflects their pain [81,82]. In the present study, we used face scales [56,57,58,60,83,84,85] in addition to the SCASS. Although visual analog, numerical, verbal, and face pain scales and the SCASS are all accurate and recommended for assessing dentinal hypersensitivity, the SCASS demonstrated the highest sensitivity and specificity and should be considered the preferred scale [86]. In addition, the SCASS has been effectively used in pediatric populations [16,17]. We have found that hypersensitivity is significantly reduced when using CPP-ACP products compared to fluoride varnish. Separate analyses on their mean effects showed this trend across various CPP-ACP combinations, with a more significant mean reduction than fluoride varnish.

MIH enamel is characterized by a hardened surface layer that forms over both intact and degraded lesions, which may limit deeper mineralization of apparently intact lesions [13]. This may explain the seemingly more relevant effect of CPP-ACP on hypersensitivity than on remineralization, as released calcium and phosphate ions can precipitate into the open dentinal tubules and form a protective superficial layer of calcium phosphate that prevents external stimuli from reaching the nerves, but not from penetrating a deeper level for proper remineralization of the body of the lesion. Moreover, as previously discussed, fluorescence-based tools are indirect methods for monitoring remineralization, and studies have reported conflicting results on the effectiveness of laser fluorescence (LF) as a useful method for detecting remineralization [87,88,89,90]. Minor mineral changes may not be accurately detected by LF devices [88,89,91], and research suggests that these devices are less effective in assessing outer and inner enamel, showing a stronger correlation with lesion depth than with mineral loss [92]. Therefore, these findings should be interpreted with caution.

Other materials, such as amine fluoride toothpaste, arginine and calcium carbonate paste, hydroxyapatite paste, low-level laser treatment, and silver diamine fluoride, have been initially explored in the studies included here. All of them exhibited a moderate effect on hypersensitivity, with low-level laser treatment showing the best results, followed by SDF, but only two studies could be included [35,59]. Previous studies have suggested that low-level laser therapy (LLLT) may help alleviate dentinal hypersensitivity (DH), although its effectiveness is debated, [93,94,95], with some results potentially influenced by placebo effects [96]. A recent review also found that silver diamine fluoride (SDF) at a concentration of 38% may reduce DH, but its long-term effects remain uninvestigated [97,98]. Despite promising results, further research on these agents is needed before recommending them.

### 4.1. Strengths and Limitations

This work is the first complex quantitative analysis of available strategies to manage remineralization and hypersensitivity in MIH. Our analyses were performed according to lesion severity, where possible. Furthermore, this systematic review and meta-analysis employed rigorous methods, adhering to the PRISMA guidelines. Validated diagnostic criteria (EAPD, Weerheijm, and Ghanim) ensured the consistency of MIH diagnosis across studies. The large sample size, particularly in hypersensitivity assessments, increases the robustness of the findings. By conducting subgroup analyses, we were able to provide a clearer picture of how different non-invasive treatments function across various severities of MIH lesions, offering valuable insights for clinicians.

Our study faced several limitations. First, the heterogeneity in study designs, follow-up periods, interventions, and outcome measures (e.g., variations in scales for hypersensitivity) limited our ability to compare the studies directly. This was particularly relevant in remineralization assessments, where different methodologies made defining a consistent control group challenging. For hypersensitivity, different scales and measurement methods also impacted the comparability of the results.

Second, despite the 15 studies, many were judged to have moderate or high risk of bias, particularly in domains such as deviations from intended interventions. This high risk of bias affected confidence in the pooled results, particularly in the hypersensitivity outcome assessments, where five studies were at serious risk of bias. Non-randomized studies similarly showed serious or moderate risk of bias, particularly in the selection of participants.

In addition, there needs to be more possibility for long-term data analysis, restricting conclusions on the long-term efficacy and durability of interventions. Finally, specific promising interventions, such as ozone therapy, were underrepresented in the data, limiting our ability to draw definitive conclusions on their efficacy, which affects robustness in the recommendations for hypersensitivity and remineralization.

### 4.2. Implications for Practice

Scientific results need to be translated rapidly into clinical practice [99,100,101,102]. In terms of the socio-psychological effects of hypersensitivity and the higher susceptibility to caries and PEB in pediatric patients with MIH, early management using desensitization agents and fluoridated toothpaste is recommended, especially for mild cases, to reduce discomfort and prevent further enamel breakdown. For severe lesions, individualized treatment plans, including preventive agents like CPP-ACP, should be considered alongside symptom management approaches. Ensuring that clinicians choose the most effective yet accessible options may improve patient outcomes while controlling costs.

### 4.3. Implications for Research

More robust, randomized controlled trials with uniform outcome measures and follow-up times are needed to better assess the long-term impact of non-invasive treatments. Standardized tools for measuring hypersensitivity, and including lesion characteristics such as severity, extent, and color, would allow for more consistent and reliable intervention comparisons.

Moreover, the role of novel therapies such as low-level laser treatment and SDF should be explored in more detail, as they were underrepresented in the current meta-analysis. Future studies with larger sample sizes and extended follow-up periods are needed to determine novel treatments’ long-term efficacy and practicality. The moderate quality of evidence calls for well-designed research to establish evidence-based clinical protocols for the management of MIH.

## 5. Conclusions

Although CPP-ACP did not show a significantly superior remineralization effect compared to fluoride-based treatments, fluorescence based-methods were more effective than fluoride in reducing hypersensitivity. Furthermore, agents such as CaGP, SDF, and low-level laser treatment had mild-to-moderate effects on remineralization and hypersensitivity management. The current evidence base, however, is limited and heterogeneous, highlighting the need for high-quality, long-term RCTs, focusing on treatment combinations and lesion severity, to better generalize these results and guide the management of MIH.

## Figures and Tables

**Table 1:** Basic characteristics of the studies included in the systematic review and meta-analysis.

Author (Year), Country	Study Design	Sample Characteristics			MIH Severity (Criteria)	Outcome Measures	Interventions	Follow-Up Timepoints
		Age Range (Mean ± SD)	Patients (Drop-Out)	Teeth				
Al-Nerabieah et al. (2024), Syria	RCT (split-mouth)	6–9 (7.6 ± 1.4)	100 (0)	200	Mild (EAPD)	Caries development and activity: DMFT/ICDAS, post-eruptive breakdown; Hypersensitivity Airblast: SCASS	- CPP-ACPFV (Mi Varnish) - 38% SDF	T0: Baseline T1: 3 months T2: 6 months T3: 9 months T4: 12 months
Bardellini et al. (2024), Italy	RCT	6–14 (9.5 ± 2.7)	39 (0)	159	Mild and severe (EAPD)	Hypersensitivity Airblast: VAS (WBFPS)	- CPP-ACFP (MI Paste Plus) + sham light therapy - Placebo mousse + PBMT - CPP-ACFP + PBMT	T0: Baseline T1: After session T2: 7 days T3: 14 days T4: 4 weeks
Ballikaya et al. (2022) ^a^ and Erbas et al. (2024) ^b^, Turkey	RCT (split-mouth)	6–13 (8.8 ± 1.5)	48 (3)	106	Mild (EAPD)	Hypersensitivity Airblast: SCASS; Clinical performance of sealants (USPHS)	- SDF; - SDF + ART (HVGIC)	T0: Baseline ^(a)^ T1: 1 month ^(a)^ T2: 6 months ^(a)^ T3: 12 months ^(a)^ T4: 18 months ^(b)^ T5: 24 months ^(b)^ T6: 36 months ^(b)^
Bakkal et al. (2017), Turkey	Prospective cohort	7–12 (9.9 ± 1.6)	54 (0)	38	Mild and severe (N/R)	Remineralization: Laser fluorescence (LF)	- 10% CPP-ACP - CPP-ACFP (10% CPP-ACP + 0.2% NaF—900 ppm)	T0: Baseline T1: 30 days
Bekes et al. (2017), Germany	Non-randomized trial	6–14 (8.2 ± 1.9)	19 (3)	56 (12)	Mild and severe (EAPD)	Hypersensitivity Airblast: SCASS; Tactile: WBFS	- 8% arginine and calcium carbonate paste	T0: Baseline T1: 1 week T2: 2 weeks T3: 4 weeks T4: 8 weeks
Biondi et al. (2017), Argentina	Prospective cohort	6–17 (N/R)	56 (0)	92	Mild and severe (N/R)	Remineralization: Laser fluorescence (LF)	- 5% NaF varnish - 10% CPP-ACP crème - 5% NaF varnish + tricalcium phosphate (TCP)	T0: Baseline T1: Day 15 T2: Day 30 T3: Day 45
Ehlers et al. (2021), Germany	RCT	6–11 (Group A 8.6 ± 1.5; Group B: 8.4 ± 1.2)	21 (7)	48	Mild and severe (N/R)	Hypersensitivity Airblast (SCASS); Tactile (WBFS); Oral hygiene (API); Rating of toothpaste taste (VAS)	- 10% hydroxyapatite paste - Amine fluoride toothpaste (1400 ppm)	T0: Screening T1: Baseline T2: 28 ± 3 days T3: 56 ± 3 days
Fütterer et al. (2019), Germany	Prospective cohort	3–15 (8.5 [N/R])	78 (0)	218	Mild and severe (EAPD)	Hypersensitivity Airblast (SCASS); Tactile (WBFS)	- Fluoride varnish - Fissure sealant - Fillings - Stainless steel crown (SSC)	T0: Before T1: After (varying from 7 to 488 days)
Muniz et al. (2019), Brazil	RCT	8–12 (8.9 ± 2.1)	66 (6)	214	Mild and severe (EAPD)	Hypersensitivity Airblast (Pimenta-modified VAS)	- Laser - 5% NaF varnish - 5% NaF varnish and laser	T0: Baseline T1: 1 week T2: 2 weeks T3: 3 weeks T4: 4 weeks
Olgen et al. (2022), Turkey	RCT	6–9 (7.7 ± [N/R])	67 (18)	90	Mild and severe (Weerheijm et al.)	Remineralization: Laser fluorescence (LF); ICDAS	- 5% NaF varnish - 10% CPP-ACP crème - CPP-ACFP (10% CPP-ACP + 0.2% NaF—900 ppm) - Fluoride toothpaste (1450 ppm)	T0: Baseline T1: 3 months T2: 6 months T3: 9 months T4: 12 months T5: 15 months T6: 18 months T7: 21 months T8: 24 months
Özgul et al. (2018), Turkey	RT	7–12 (N/R)	33 (0)	92	Mild (N/R)	Hypersensitivity: Cold stimuli (VAS)	- 5% NaF varnish - Ozone + 5% NaF varnish - 10% CPP-ACP paste - Ozone + 10% CPP-ACP paste - CPP-ACFP - Ozone + CPP-ACFP	T0: Baseline T1: 4 weeks T2: 12 weeks
Pasini et al. (2018), Italy	Prospective randomized trial	8–13 (N/R)	40 (0)	40	Mild and severe (Weerheijm et al., 2003)	Hypersensitivity: Airblast (SCASS); Mechanical stimulus (VAS)	- Fluoride toothpaste (1000 ppm) - 10% CPP-ACP paste	T0: Baseline T1: 4 months
Restrepo et al. (2016), Brazil +	RT	9–12 (10.2 ± 1.1)	51 (0)	51	Mild and Severe (EAPD)	Remineralization: Quantitative light-induced fluorescence imaging (QLF)	- 4 × applications 4% NaF varnish - Fluoride Toothpaste (1450 ppm)	T0: Baseline T1: 4 weeks
Sezer et al. (2022), Turkey	RCT (crossover)	8–12 (9.2 ± 1.4)	22 (0)	162	Mild and severe (Weerheijm et al., 2003)	Remineralization: Laser fluorescence (LF)	- CPP-ACFP (MI Paste Plus^TM^) - CaGP (R.O.C.S.^®^)	T0: Baseline 2 weeks lead-in T1: 12 weeks 2 weeks washout T2: 12 weeks
Sezer and Kargul, (2022), Turkey	RCT	8–12 (9.3 ± 1.4)	53 (0)	401	Mild and severe (Ghanim et al., 2015)	Remineralization: Laser fluorescence (LF)	- CaGP (R.O.C.S.^®^) - CPP-ACFP (MI Paste Plus^TM^) - Fluoride toothpaste (1450 ppm)	T0: Baseline T1: 1 month T2: 3 months
Singh et al. (2021), India	RT (pilot study)	8–14 (N/R)	30 (0)	30	Mild and severe (Weerheijm et al., 2003)	Remineralization: Laser fluorescence (LF)	- 5% NaF varnish - 10% CPP-ACP paste	T0: Baseline T1: 15 days

+ Study was not included in the quantitative analysis due to a different outcome measure (QLF) used from the other studies (LF). Legend: a: Ballikaya et al. (2022) ^(a)^; ART: atraumatic restorative treatment; b: Erbas et al. (2024) ^(b)^; CaGP: calcium glycerophosphate; CPP-ACP: casein phosphopeptide–amorphous calcium phosphate; CPP-ACFP: casein phosphopeptide–amorphous calcium fluoride phosphate; CPP-ACPFV: casein phosphopeptide–amorphous calcium phosphate fluoride varnish; DMFT: decayed, missing, and filled teeth; EAPD: European Academy of Pediatric Dentistry; HVGIC: high-viscosity glass ionomer cement; ICDAS: International Caries Detection and Assessment System; LF: laser fluorescence; MIH: molar–incisor hypomineralization; NaF: sodium fluoride; N/R: not reported; PBMT: photobiomodulation therapy; QLF: quantitative light-induced fluorescence; RCT: randomized controlled trial; RT: randomized trial; SCASS: Schiff Cold Air Sensitivity Scale; SDF: silver diamine fluoride; USPHS: United States Public Health Service; VAS: visual analog scale; WBFS: Wong–Baker Pain Rating Scale.

## Data Availability

All data generated or analyzed during this study are included in this article, the Supplementary Material files, and in the full-text articles included in this meta-analysis. Further inquiries can be directed to the corresponding author (G.V.).

## Supplementary Materials

The following supporting information can be downloaded at https://www.mdpi.com/article/10.3390/jcm13237154/s1: Table S1: PRISMA 2020 Checklist; Table S2: Search strategies (search performed on 8 July 2024); Table S3: Description of severity level according to the EAPD criteria (described by Lygidakis et.al, 2022 [26]); Table S4: List of studies excluded during full-text selection; Figure S1: Risk-of-bias tools used for the included studies: (A) ROBINS-I tool for interventional, non-randomized controlled trials, and (b) RoB 2.0 tool for interventional, randomized controlled trials; Figure S2: Forest plot of mean change from baseline values of different agents used for hypersensitivity management, measured on the VAS pain scale (0 to 10), for MIH lesions.
